# Activation of Interleukin-1β Release by the Classical Swine Fever Virus Is Dependent on the NLRP3 Inflammasome, Which Affects Virus Growth in Monocytes

**DOI:** 10.3389/fcimb.2018.00225

**Published:** 2018-07-02

**Authors:** Shuangqi Fan, Jin Yuan, Shaofeng Deng, Yuming Chen, Baoming Xie, Keke Wu, Mengjiao Zhu, Hailuan Xu, Yunzhen Huang, Jiongfeng Yang, Yangyi Zhang, Jinding Chen, Mingqiu Zhao

**Affiliations:** Department of Microbiology and Immunology, College of Veterinary Medicine, South China Agricultural University, Guangzhou, China

**Keywords:** classical swine fever virus, Flavivirus, NLRP3 inflammasome, interleukin-1β, monocyte, innate immune response

## Abstract

Classical swine fever virus (CSFV) is a classic Flavivirus that causes the acute, febrile, and highly contagious disease known as classical swine fever (CSF). Inflammasomes are molecular platforms that trigger the maturation of proinflammatory cytokines to engage innate immune defenses that are induced upon cellular infection or stress. However, the relationship between the inflammasome and CSFV infection has not been thoroughly characterized. To understand the function of the inflammasome response to CSFV infection, we infected porcine peripheral blood monocytes (PBMCs) with CSFV. Our results indicated that CSFV infection induced both the generation of pro-interleukin-1β (pro-IL-1β) and its processing in monocytes, leading to the maturation and secretion of IL-1β through the activation of caspase 1. Moreover, CSFV infection in PBMCs induced the production and cleavage of gasdermin D (GSDMD), which is an inducer of pyroptosis. Additional studies showed that CSFV-induced IL-1β secretion was mediated by NLRP3 and that CSFV infection could sufficiently activate the assembly of the NLRP3 inflammasome in monocytes. These results revealed that CSFV infection inhibited the expression of NLRP3, and knockdown of NLRP3 enhanced the replication of CSFV. In conclusion, these findings demonstrate that the NLRP3 inflammasome plays an important role in the innate immune response to CSFV infection.

## Introduction

Classical swine fever virus (CSFV) is the etiological agent of classical swine fever (CSF), which is characterized by a hemorrhagic syndrome and immunosuppression in piglets and is responsible for heavy economic burdens in many countries (Kleiboeker, 2002; Lohse et al., 2012). CSFV belongs to the genus *Pestivirus* within the family *Flaviviridae* and has a small, enveloped single-stranded positive-sense 12.3 kb RNA genome. The genome includes a long open reading frame (ORF) that encodes a 3,898 amino acid precursor polyprotein, which is initially translated (Becher et al., 2003). Cell-specific and virus-associated proteases cleave the precursor protein as it is processed to form four CSFV structural proteins (C, Erns, E1, and E2) and eight non-structural proteins (Npro, P7, NS2, NS3, NS4A, NS4B, NS5A, and NS5B) (Li et al., 2017). While CSFV has not been observed to cause cytopathic effects in susceptible cells (Johns et al., 2010), studies have shown that CSFV is able to infect dendritic cells (DCs), macrophages, and vascular endothelial cells. CSFV infection induces cellular immunosuppression, which compromises the host immune system (Chen et al., 2012; Dong and Tang, 2016).

Innate immunity is considered the first line of defense to distinguish between “self” and “non-self” (Gallo and Hooper, 2012). The inflammasome, a component of the innate immune response (Dolasia et al., 2017; Seveau et al., 2017), is a macromolecular complex that includes the pro-inflammatory protease caspase 1, the recruitment linker molecule ASC, and NLR or PYHIN family members and has been widely studied in recent years (Lupfer and Kanneganti, 2012). So far, 23 NLR genes have been identified in humans, and at least 34 NLR genes have been identified in mice (Inohara and Nunez, 2001; Harton et al., 2002; Ting et al., 2008). However, the function of most NLRs remains unknown. Currently, the NLRP3 inflammasome is the most thoroughly studied type of inflammatory complex; it is involved in the development of many human diseases and is activated upon host exposure to whole pathogens and environmental irritants (Schroder and Tschopp, 2010).

NLRP3 has been widely shown to be activated during infections with pathogenic microbes by interleukin-1β (IL-1β) (Ali et al., 2017). IL-1β, which is a key cytokine, is associated with both acute and chronic inflammation and with viral disease (Negash et al., 2013). During viral infection, IL-1β production is induced by cellular sensing of pathogen-associated molecular patterns (PAMPs) (Vance et al., 2009). Two signals are required to produce activated IL-1β. The first signal activates NF-kB in stimulated cells and increases the expression of IL-1β mRNA. The second signal activates an NLR to promote the cleavage of caspase 1, which is involved in the processing of pro-IL-1β into a biologically active and secreted cytokine (Franchi et al., 2009).

Caspase 1 is a cysteine protease whose auto-cleavage produces the activated caspase-1p10/p20 tetramer, which is regulated by inflammasomes (Martinon and Tschopp, 2007).

Recent studies have shown that certain flaviviruses, including hepatitis C virus (HCV), West Nile virus (WNV), and Japanese encephalitis virus (JEV), induce IL-1β production by the NLRP3 inflammasome (Kaushik et al., 2012; Ramos et al., 2012).

While earlier work had revealed that CSFV infection induces the expression and activation of IL-1β in swine macrophages (Kaushik et al., 2012; Ramos et al., 2012), the precise mechanism of inflammasome assembly that is triggered by CSFV infection remains unclear. Moreover, activation of caspase 1 can cause pyroptosis, a form of cell death that is different from apoptosis. Pyroptosis is a proinflammatory form of regulated cell death that is dependent on caspase 1 activation (Chen et al., 2016).

Caspase 1, following activation by various inflammasomes, cleaves gasdermin D (GSDMD) to produce an N-terminal cleavage product (GSDMD-N), which is a critical component of pyroptosis (Chang et al., 2013). Following the production of GSDMD-N, cells undergoing pyroptosis develop DNA damage, chromatin condensation, pore formation in membranes that stain positively as dead cells, cell lysis, and the release of pro-inflammatory cytokines (Wree et al., 2014; Shi et al., 2015).

Leukopenia is a typical hallmark of clinical CSF, which likely leads to the immunosuppression caused by CSFV infection. Apoptosis has generally been considered the cause of leukocyte death (Kepka et al., 2014). However, the mechanisms of cell apoptosis that are induced by CSFV infection are still confusing and undefined.

Recent studies have shown that some characteristics of pyroptosis are similar to apoptosis, with the cells incurring DNA damage and staining positively in TUNEL assays, staining positively with annexin V, and forming spherical membrane-bound structures (Miao et al., 2011; Lin et al., 2013). These similarities inspired us to investigate these “apoptotic cells” and examine whether pyroptosis is a component of the lymphocytopenia process caused by CSFV infection. To our knowledge, few studies have investigated the relationship between CSFV infection and pyroptosis.

Because CSFV has a partial tropism for immune-related organs and immune cells, and pyroptosis is observed primarily in monocytes, macrophages, and dendritic cells, monocytes were chosen for investigating which type of inflammasome is assembled and whether pyroptosis occurs after CSFV infection. In the current study, we investigated the relationship among inflammasomes, pyroptosis, and CSFV infection in monocytes. By perturbing the inflammasome pathway with regulators and short hairpin RNA (shRNA) molecules and then monitoring the formation of inflammasomes, we determined that CSFV infection induced IL-1β production through the NLRP3 inflammasome. Furthermore, by inhibiting NLRP3 in monocytes, we found that CSFV-induced pyroptosis promoted CSFV replication, suggesting that the NLRP3 inflammasome exerts anti-CSFV activity.

## Materials and methods

### Virus and antibodies

The virulent CSFV strain Shimen was maintained in our laboratory and the virus titers were determined using methods previously described (Pei et al., 2014). Primary antibodies used in the study included mouse monoclonal anti-CSFV E2 (JBT, 9011), mouse polyclonal anti-CSFV Npro (kindly provided by Dr. Xinglong Yu, Veterinary Department, Hunan Agricultural University, China), rabbit polyclonal anti-caspase-1 p10 (sc-514; Santa Cruz Biotechnology), mouse monoclonal anti-IL-1β (MP425; Thermo Fisher Scientific), rabbit polyclonal anti-IL-1β (ASC0912; Invitrogen), mouse monoclonal anti-GAPDH (AG019; Beyotime), mouse monoclonal anti-tubulin (AT819; Beyotime), rabbit monoclona anti-NLRP3 antibody (bs-23723R; Bioss), rabbit monoclona anti-ASC(bs-6741R; Bioss), rabbit polyclonal anti-Gasdermin D/DFNA5L(bs-14287R; Bioss), rabbit monoclonal anti-RIG-I antibody (D33H10; Cell Signaling Technology), and rabbit monoclonal anti- Caspase-1 antibody (D7F10; Cell Signaling Technology).The secondary antibodies included horseradish peroxidase (HRP)-conjugated goat anti-mouse IgG (BS12478; Bioworld), HRP-conjugated goat anti-rabbit IgG (BS13278; Bioworld), Dylight 488 goat anti-mouse IgG (E032210; EarthOx Life Sciences).

### Monocyte isolation and infection

Citrated blood was obtained from specific pathogen-free (SPF) pigs (60 days). Peripheral blood mononuclear cells (PBMCs) were isolated from the citrated blood samples using Ficoll-Hypaque (density 1.077 g/L, TBD Sciences, Tianjin, China) and centrifugation at 800 × g for 25 min. Contaminating erythrocytes were lysed by a single treatment with NH_4_Cl buffer (0.15 M NH_4_Cl, 10 mM NaHCO_3_, pH 7.4) for 5 min. PBMCs were washed three times using phosphate-buffered saline (PBS) and pelleted at 500 × g for 10 min. Monocytes were enriched by adherence (2 h at 37°C) in phenol-red-free Dulbecco's modified Eagle medium (DMEM; Life Technologies, Basel, Switzerland) containing 10% fetal bovine serum (FBS; Life Technologies), 100 U/mL penicillin, and 100 μg/mL streptomycin. Monocytes were infected with CSFV at the indicated multiplicity of infection (MOI) at 37°C. The inoculum was removed, the cells were washed three times with PBS, and the cells were cultured in fresh DMEM supplemented with 2% FBS.

### Caspase 1 activity assays

The caspase 1 activity was measured using a Caspase 1 Activity Kit (C1101; Beyotime, Shanghai, China) according to the manufacturer's instructions. Caspase 1 catalyzes the production of yellow p-nitroaniline (pNA) from the substrate acetyl-Tyr-Val-Ala-Asp p-nitroanilide (Ac-YVAD-pNA). The cells were harvested and lysed on ice for 15 min, centrifuged at 16,000 × g for 10 min, and the supernatant (50 μg protein) was mixed with synthetic tetrapeptide Ac-YVAD-pNA and incubated at 37°C for 2 h. The absorbance of the pNA was spectrophotometrically determined at 405 nm using a 96-well plate reader (BioTek, Santa Barbara, CA, USA).

### Immunofluorescence assay (IFA)

The infected monocytes were fixative, permeabilized, and subsequently immunostained by incubating the cells with primary antibody anti-CSFV E2 mAb, followed by incubation with the secondary antibody fluorescein isothiocyanate (FITC)-conjugated anti-mouse IgG. After washing with PBS three times, cells were examined using an LSM 510 Meta confocal laser scanning microscope (Carl Zeiss, Gottingen, Germany).

### Enzyme-linked immunosorbent assay

An IL-1β ELISA was carried out according to the manufacturer's protocol (Raybiotech).

### Western blot analysis

Western and immunoprecipitation (IP) cell lysis buffer (P0013; Beyotime) was chilled on ice, and phenylmethylsulfonyl fluoride (PMSF) purchased from Beyotime (ST506) was added to a final concentration of 1 mM prior to use. Cell lysates were recovered from CSFV-stimulated or control samples via cell lysis for 45 min on ice. The lysate was centrifuged at 13,000 × g for 25 min at 4°C. Next, the supernatant was collected and the protein concentration measured using a BCA protein assay kit (23227; Thermo Fisher Scientific). Protein samples were boiled for 5 min in 5 × sodium dodecyl sulfate polyacrylamide gel electrophoresis (SDS-PAGE) loading buffer. Equal amounts of protein for each sample were separated using 12% SDS-PAGE gels and transferred onto polyvinylidene fluoride (PVDF) membranes (IPVH00010; Millipore). The PVDF membranes were blocked with 5% skim milk dissolved in PBST at 37°C for 2 h. Next, the PVDF membranes were incubated with primary antibodies at 4°C overnight, and the corresponding HRP-conjugated secondary antibodies were added at 37°C for 1 h, at appropriate dilutions (1:1,000). An ECL Plus kit (P0018; Beyotime) and chemiluminescence imaging system (Fine-do X6, Tanon) were used for detecting and imaging the protein bands. Image-Pro Plus 6.0 software (Media Cybernetics) was used to quantitate the protein in the blots according to the user's guide.

### Lentivirus production and transduction

The shRNAs for swine NLRP3 used in the study are listed in Table 1. The shRNA plasmids (pLenti-shNLRP3) were transfected into HEK293T cells together with pMDLg, pRSV-rev, and pCMV-VSV-G using Lipofectamine 3000 (Life Technologies). The ratio of pLenti-shNLRP3, pMDLg, pRSV-rev, and pCMV-VSV-G was 2:1:1:1, respectively. Culture supernatants were harvested at 24 h post-transfection (pt) and 48 h pt and were centrifuged at 4,500 × g for 20 min at 4°C to remove cellular debris. The recovered viruses were concentrated using an Amicon Ultra-15 centrifugal filter unit with an Ultracel-10 membrane (Millipore). The viruses were resuspended in RPMI-1640 medium, and the viral titers were determined using HEK293T cells. Monocytes were transduced with the same amounts of lentiviral particles encoding non-targeting control or gene-specific shRNA in the presence of 8 μg/mL polybrene. After 48 h, monocytes were infected with CSFV at 1 MOI. After another 12 h, the shRNA knockdown efficiency of the target protein in lentivirus-transduced cells was assessed by western blot analysis. The concentrations of IL-1β were measured by ELISA.

**Table 1:** Sequences of shRNA-NLRP3.

**NLRP3**	**Sequence 5′-3′**
1	GGTGA CCTCATATGA CTAA
2	GCATC TATTCTGCAA GCTA
Scrambled	CGAAT CCTACAAAGC GCGC

### Cell death assay

Cell death was assessed by EthD-III/calcein AM staining (Viability/Cytotoxicity Assay Kit, Biotium, USA). EthD-III/calcein AM staining was also used for the cell death assay. The AM stain produced a bright green fluorescence in live cells. EthD-III entered dead cells, thereby producing red fluorescence in dead cells. Cells were simultaneously stained with 2 mM calcein AM and 4 mM EthD-III for 45 min at room temperature. Samples were analyzed using flow cytometry (BD FACSVerse, BD Biosciences, USA). The percentage of dead cells was determined by counting the ratio of red-positive cells to blue-positive cells.

### Statistical analysis

Statistical analysis using unpaired Student's *t*-test was performed using Graph Pad Prism 5 software.

## Results

### CSFV stimulated the maturation of IL-1β in monocytes

**Figure 1:**
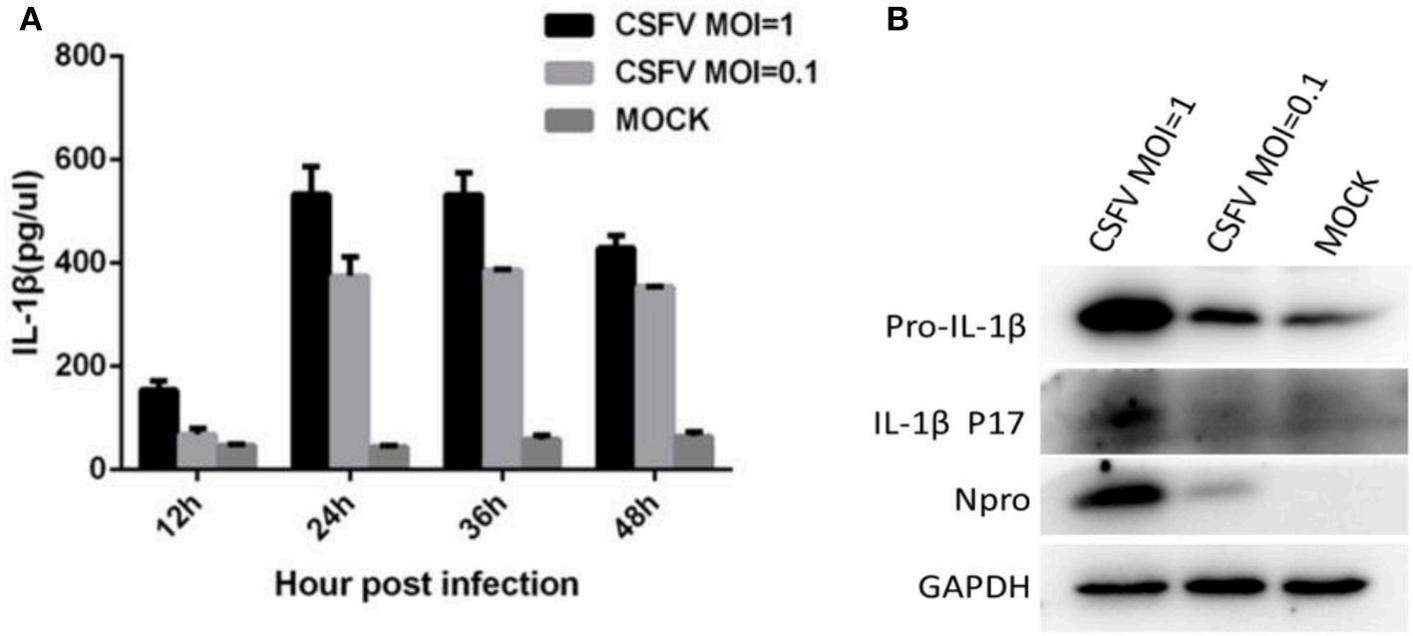
CSFV infection induced the maturation and secretion of IL-1β. **(A)** PBMCs were mock treated or infected with CSFV at a MOI of 1 or 0.1 for different lengths of time. The secretion of IL-1β was detected using an IL-1β-specific ELISA kit at different time points. **(B)** The expression of pro-IL-1β and the subunit p17 were identified by western blot analysis of cell lysates following CSFV infection (MOI of 1 or 0.1) at 24 h pi. Npro protein was detected by western blotting using an anti-CSFV Npro antibody for confirmation of CSFV infection. GAPDH was used as an internal loading control.

We evaluated the ability of CSFV to induce the secretion of IL-1β. Monocytes were infected with CSFV at MOIs of 1 or 0.1. Cell supernatant and lysate samples were harvested at different time points. The cell supernatant was used to assess the release of mature IL-1β using an IL-1β-specific ELISA kit. As shown in Figure 1A, IL-1β secretion significantly increased after CSFV infection in a dose-dependent manner compared with that from the mock-infected cells. At 24 h post-infection (pi), the level of IL-1β reached its highest peak. Western blot analysis showed that the expression of pro-IL-1β was increased, and the p17 subunit was detected in the CSFV-infected cells (Figure 1B).

### CSFV-induced caspase 1 activation and caspase 1 dynamics were necessary for operation of IL-1β in CSFV-infected monocytes

**Figure 2:**
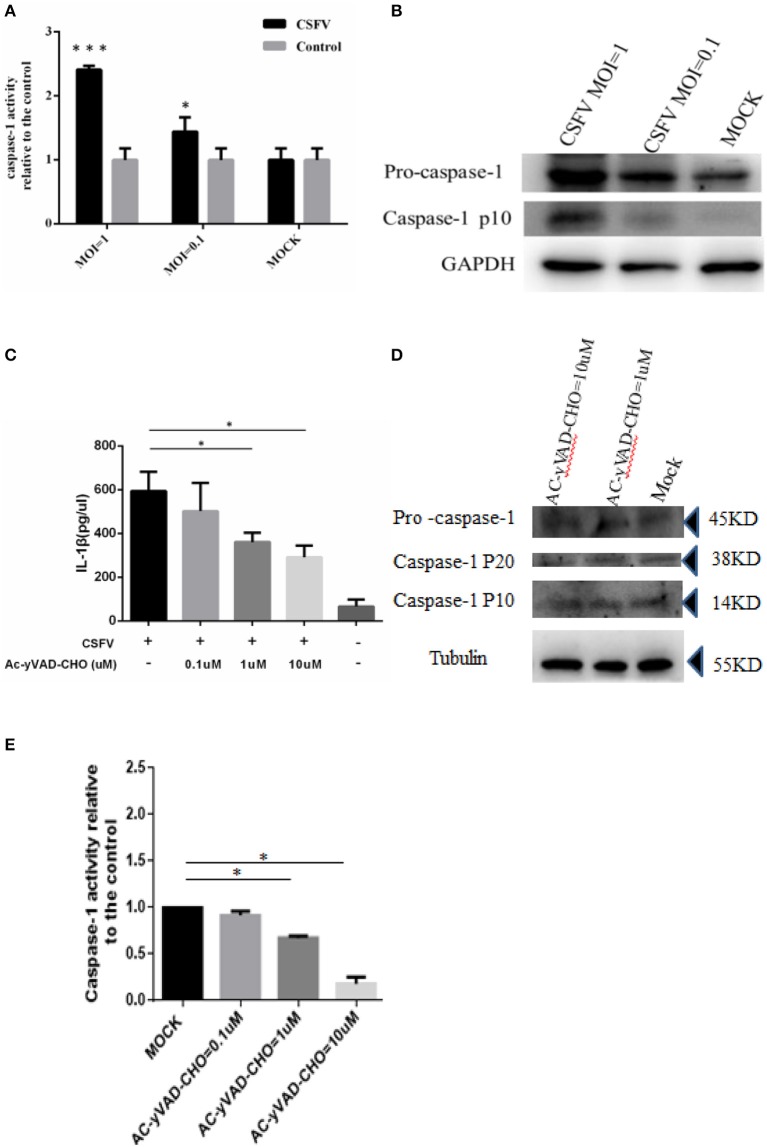
Activation of IL-1β induced by CSFV infection was dependent on caspase 1. **(A)** The caspase 1 was activated by CSFV infection. PBMCs were mock treated or infected with CSFV at a MOI of 1 or 0.1 for 6 h. **(B)** The expression of pro-caspase 1 and the cleavage of caspase-1p10 protein were identified by western blot analysis following CSFV infection (MOI of 1 or 0.1). **(C)** Following treatment with the caspase 1 inhibitor Ac-yVAD-CHO, the activation of IL-1β induced by CSFV was inhibited. **(D)** The expression of caspase 1 precursor and the subunit p20/P10 proteins were not affected by treatment with AC-yVAD-CHO. **(E)** Caspase 1 activity was dose-dependently inhibited by AC-yVAD-CHO, and CSFV activated caspase 1 activity at an MOI of 1. *P < 0.05; ***P < 0.001. *P*-values were calculated using an One-way ANOVA test.

In response to inflammatory agents, the immature pro-IL-1β is cleaved into mature IL-1β, which was required for the activation of caspase 1 (Kanneganti et al., 2006). After synthesis, caspase exists in the form of zymogen and is inactive. After the cell obtains the initial apoptotic signal, caspase 1 is cleaved at its C-terminus to become its active form and participate in apoptosis (Thornberry et al., 1992). Therefore, we investigated whether CSFV infection in monocytes could cause the activation of caspase 1. Using a caspase 1 activity assay, we determined that CSFV infection of monocytes at an MOI of 1 significantly increased the activity of caspase 1 (more than 2-fold) relative to the activity in the control group. Even at an MOI of 0.1, CSFV infection induced a greater than 1.5-fold increase in caspase 1 activity compared with that in mock-infected cells (Figure 2A). Subsequently, we quantitated levels of the pro-caspase 1 and the cleaved caspase protein by western blot analysis (Figure 2B). These results also indicated that CSFV infection had a positive effect on caspase 1 activation and expression. To determine whether activated caspase 1 was necessary for the generation of IL-1β in monocytes upon CSFV infection, we used the caspase 1 inhibitor Ac-yVAD-CHO to suppress caspase 1 activity. The cells were subsequently infected with CSFV for 6 h. Similar to the previously observed results, caspase 1 activity increased significantly. As expected, inhibiting caspase 1 activity (data not shown) induced a significant and dose-dependent decrease in IL-1β levels (Figures 2C–E). These results suggested that caspase 1 was critical for the maturation of IL-1β during CSFV infection in monocytes.

### Induction of cell death and membrane damage by CSFV infection of PBMCs was depended on the activity of caspase 1

**Figure 3:**
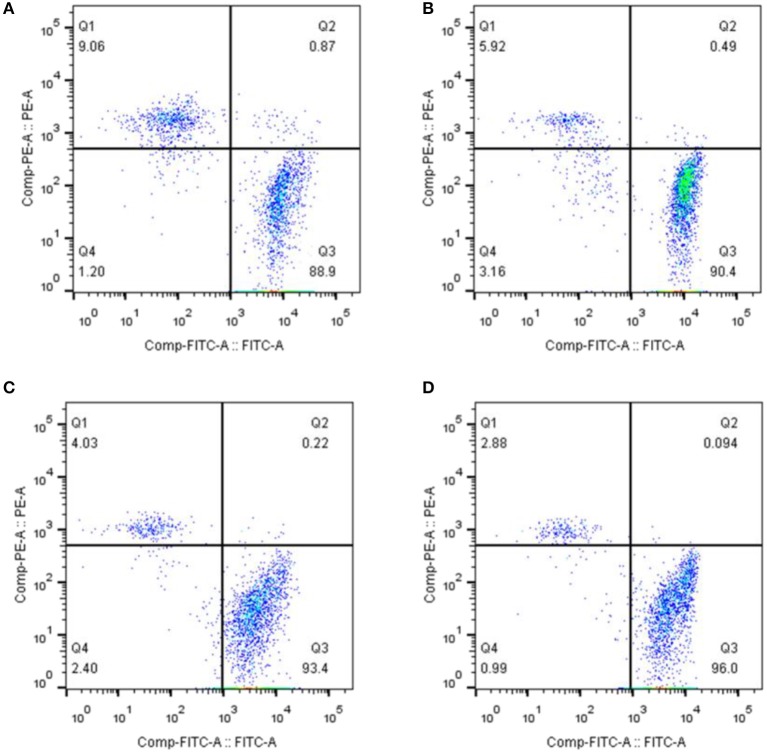
Induction of cell death and membrane damage by CSFV infection of PBMCs depended on the activity of caspase 1. **(A)** Flow cytometry was used to determine the ratio of membrane damaged peripheral blood monocytes caused by CSFV infection. PBMCs were infected with CSFV at an MOI of 1 for 24 h. **(B)** The proportion of dead PBMCs resulting from membrane damage induced by CSFV infection was affected by treatment with AC-yVAD-CHO. PBMCs were infected with CSFV at an MOI of 1 and transfected with 1 μM AC-yVAD-CHO. **(C)** PBMCs were infected with CSFV at an MOI of 1 and transfected with 10 μM AC-yVAD-CHO. The ratio of membrane damage cells was determined using Flow cytometry. **(D)** PBMCs were mock treated at 24 h.

Recent studies have shown that cell death and apoptosis are very different in their respective mechanisms of induction. Apoptosis mainly depends on the activation of caspases 3, 8, and 9, while cell death is primarily dependent on the activation of caspases 1 and 11. To further analyze whether cell death was involved in the death of lymphocytes induced by CSFV infection, we treated PBMCs with the caspase 1 inhibitor Ac-yVAD-CHO at 1 or 10 μmol/L. The cells were then infected with CSFV at an MOI of 1 and the membrane damage was detected using the Calcein-AM/EthD-III Cell Activity Assay Kit at 24 h pi. The results demonstrated that after the treatment of PBMCs with caspase 1-specific inhibitor, the proportion of live cells induced by CSFV-infected PBMCs was decreased and the proportion of dead cells with membrane damage was suppressed. The proportion of living cells decreased and the proportion of dead cells due to membrane damage increased in an inhibitor dose-dependent manner (Figure 3).

### CSFV infection of PBMCs induced the production and cleaving of GSDMD

**Figure 4:**
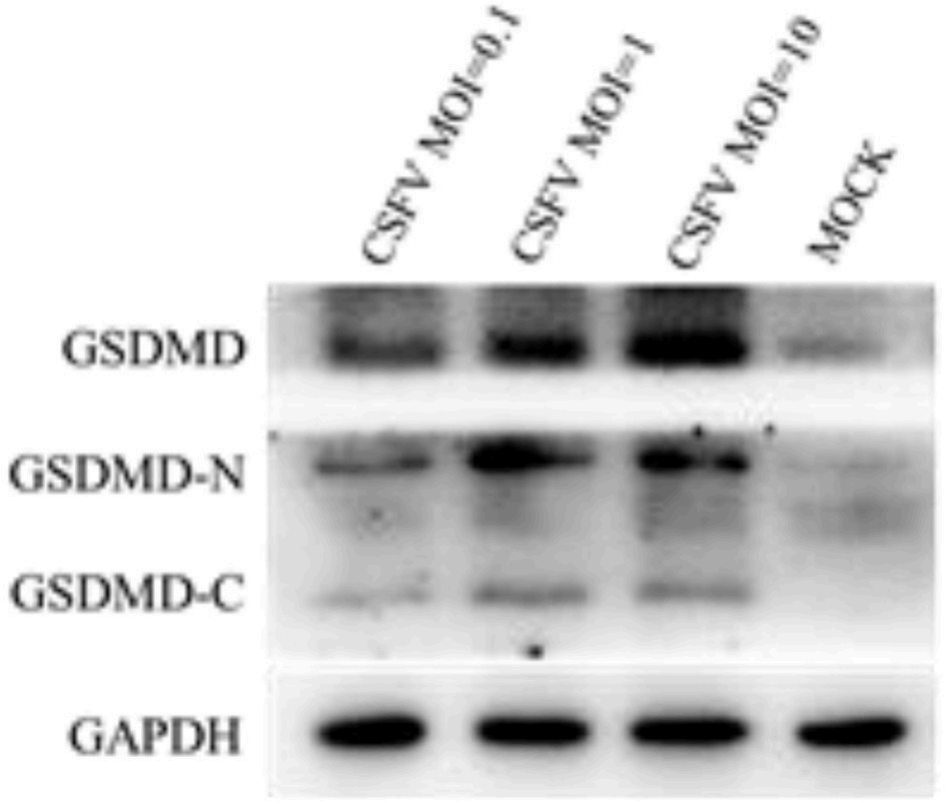
CSFV infection induced the production and cleavage of GSDMD in PBMCs. PBMCs were mock-infected or infected with CSFV (MOI of 0.1, 1, or 10). The expression levels of GSDMD, GSDMD-N, and GSDMD-C were determined by western blotting at 48 h pi. GAPDH was used as an internal loading control.

Cell pyroptosis is a more recently identified type of programmed cell death that relies on caspase 1 and is often associated with inflammatory reactions (Zhao et al., 2016). Studies have shown that the activated GSDMD-N is a functionally active part of the pyroptosis process (Aglietti and Dueber, 2017). GSDMD cleavage into an active GSDMD-N fragment is one of the criteria required for effecting pyroptosis. To determine whether CSFV infection had an effect on pyroptosis of PBMCs, we infected the PBMCs of pigs with different doses of CSFV (MOIs of 0.1, 1, and 10) and detected the expression of GSDMD and its cleavage after infection (Figure 4). The results showed that CSFV at different MOIs was able to induce GSDMD expression and catalytic processing to produce active GSDMD-N. Also, the expression level of GSDMD positively correlated with the infection level by CSFV, which indicated that CSFV infection induced pyroptosis in PBMCs.

### CSFV affects the NLRP3 inflammasome in monocytes

NLRP3 inflammasome can activate caspase 1 in response to conserved structures and threatening endogenous signals of various pathogens and can mediate the proinflammatory cytokine precursor pro-IL-1β. Also, the maturation and secretion of pro-IL-18 activate the immune defenses and play an important role in the anti-microbial response to infection, and is one of the key inflammasome molecules implicated in the identification of single-stranded RNA (ssRNA) and double-stranded DNA viruses (Kanneganti et al., 2006).

Previous studies have shown that the induction and activation of IL-1β and IL-18 are critical signatures of inflammasome activation (Malik and Kanneganti, 2017), along with the adapter protein ASC, which binding to the pyrin domain of the NLRP3 N-terminus recruits pro-caspase1 and then activates caspase1 (Stehlik et al., 2003).

**Figure 5:**
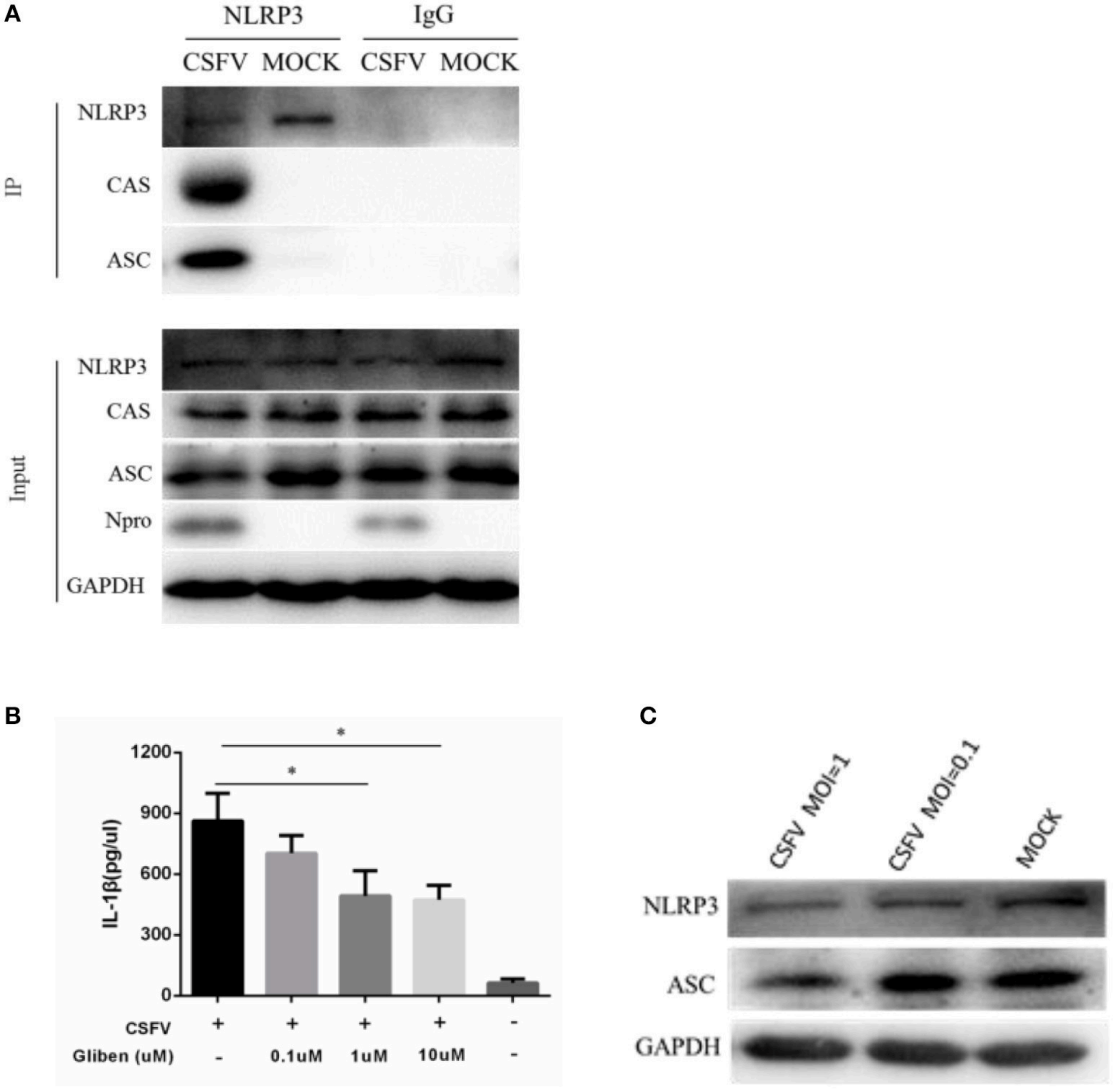
Formation of the NLRP3 inflammasome was induced in PBMCs by CSFV infection. **(A)** PBMCs were mock-infected or infected with CSFV at an MOI of 1 for 48 h. The cell lysate was collected and incubated with the anti-CAS and anti-ASC antibodies in order to immunoprecipitate (IP) caspase 1 (CAS) and ASC. Normal rabbit IgG was used as a negative control. Moreover, ASC and CAS protein levels in the lysates of whole cells were used as input controls. GAPDH was used as an internal loading control. CSFV infection was verified by immunoblotting using anti-CSFV Npro antibody. **(B)** The level of NLRP3 inflammatory complexes induced by CSFV infection was linked to ATP-dependent K+ channel activation. **(C)** CSFV infection inhibited the expression of NLRP3 and ASC. *P < 0.05. *P*-values were calculated using an One-way ANOVA test.

The mature caspase 1 proteolytically cleaves cytosolic pro-IL-1β and pro-IL-18, which are then secreted as inflammatory cytokines, which activate the inflammatory arm of the immune response to infection. However, the precise mechanism of pathogens to activate the inflammasome is unclear. To examine whether CSFV infection modulated the expression of the components of the NLRP3-dependent inflammasome, monocytes were mock-infected or infected with CSFV. Using co-immunoprecipitation methodology, we found that CSFV infection primarily affected the expression of the NLRP3 inflammatory bodies CAS and ASC (Figure 5A). Results of the western blot analysis (Figure 5B) were also consistent with the IP findings. In addition, we found that CSFV at a relatively high infection dose (MOI = 1) had an inhibitory effect on the NLRP3 and ASC proteins (Figure 5C).

### CSFV activation of interleukin-1β and its release was dependent on the NLRP3 inflammasome and RIG-I

**Figure 6:**
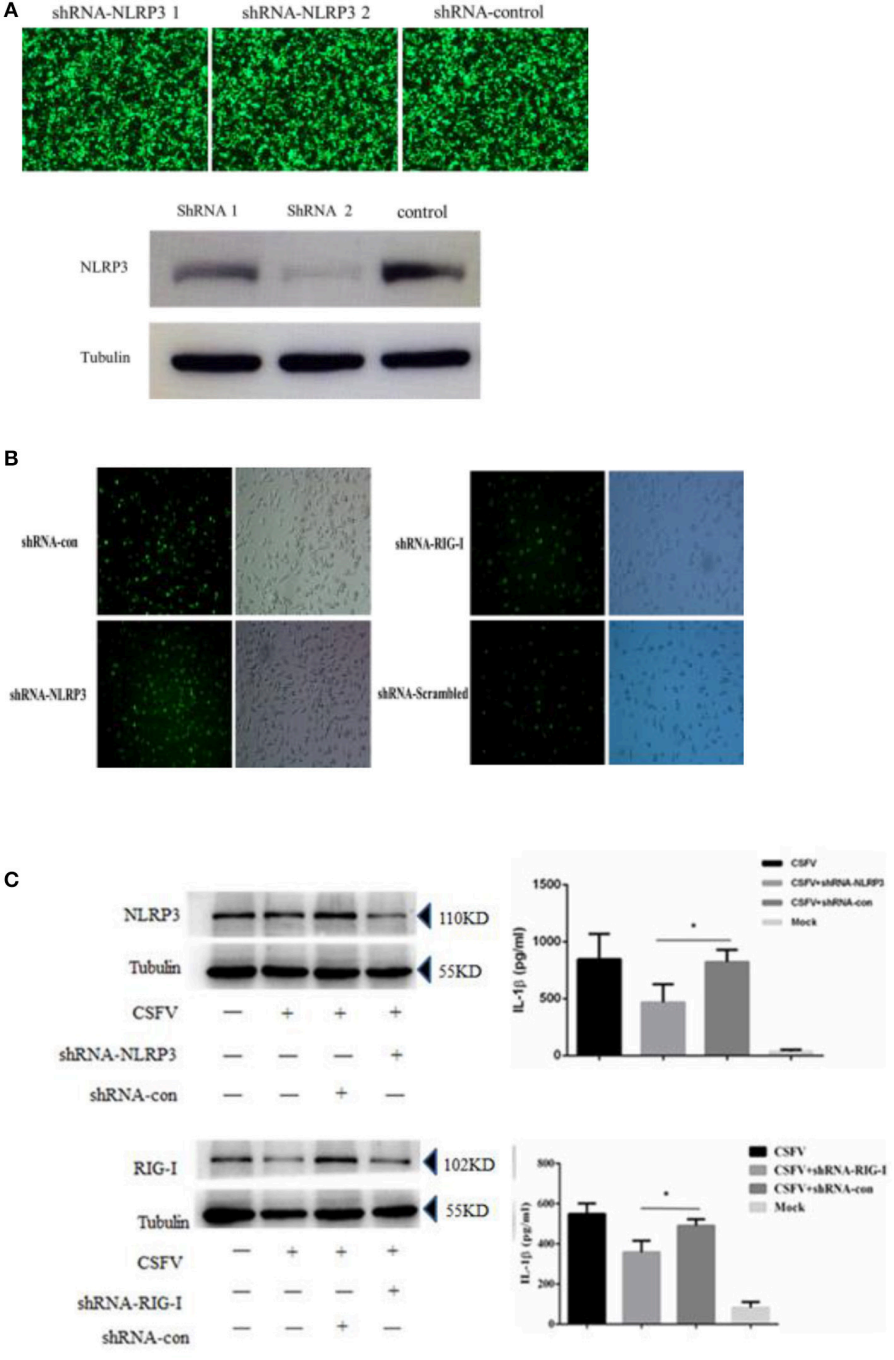
Secretion of IL-1β in PBMCs induced by CSFV infection depended on the expression of the NLRP3 and RIG-I genes. **(A)** The NLRP3 interference plasmid was highly efficient at interfering with NLRP3 expression as verified by immunofluorescence and western blotting. The images of immunofluorescence showed no difference in the transfection efficiency of the three plasmids, western blotting showed shRNA 2 had a better interference effect on NLRP3. **(B)** The rescued lentiviruses LtV-RIG-I, LtV-NLRP3, and LtV-Scrambled were successfully transfected into porcine PBMCs. **(C)** The secretion of IL-1β in porcine PBMCs induced by CSFV infection depended on expression of the NLRP3 and RIG-I genes. *P < 0.05. *P*-values were calculated using an One-way ANOVA test.

CSFV can promote the release of interleukin and inhibit the expression of the NLRP3 inflammasome protein ASC. To investigate whether the CSFV-mediated promotion of interleukin release was dependent on NLRP3, we constructed an interference plasmid using lentivirus packaging plasmids (Figure 6A), which was able to stably inhibit NLRP3 in a white blood cell model system that that could be infected by CSFV (Figure 6B). The results indicated that CSFV promotion of the release of interleukin was dependent on the functions of the NLRP3 (Figure 6C). In our previous study, we found that CSFV infection of alveolar macrophages promotes the release of RIG-I-dependent cytokines. We used interference plasmids to construct cell lines that stably inhibited the RIG-I gene and demonstrated that CSFV infection of PBMCs requires RIG-I in order to promote the release of IL-1β (Figure 6C).

### Targeting NLRP3 has a prompted effect on CSFV replication

**Figure 7:**
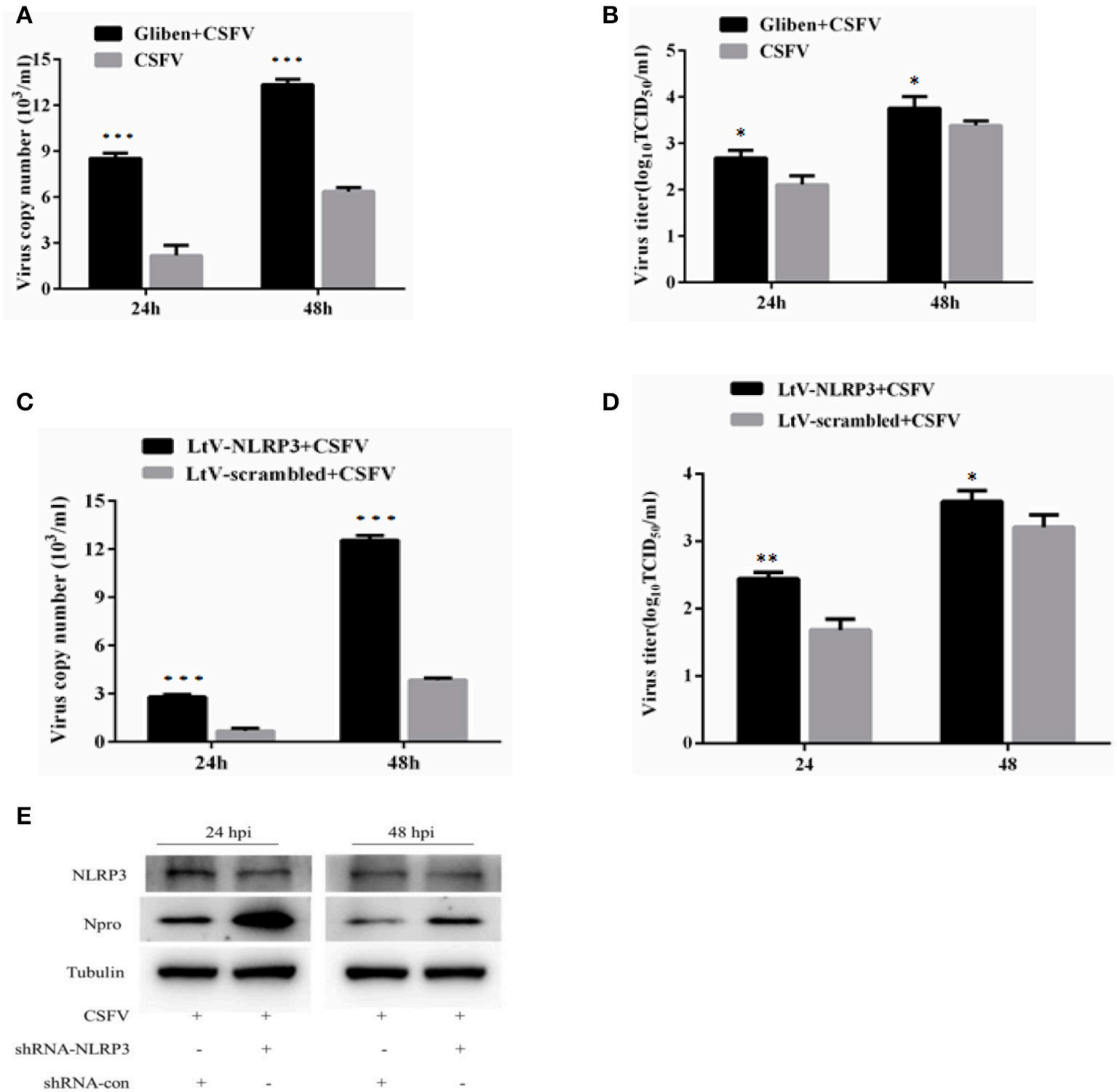
Inhibition of NLRP3 promoted the replication of CSFV. The effect of the NLRP3-specific inhibitor glibenclamide on the virus copy number **(A)** and virus titers **(B)** in CSFV-infected cells. PBMCs were treated with glibenclamide or DMSO for 24 h, followed by the cells being infected with CSFV (MOI = 1). At 24 h pi and 48 h pi, the levels of CSFV RNA were analyzed using real-time reverse transcription polymerase chain reaction (RT-qPCR) and CSFV titers were analyzed by immunofluorescence (mean ± SD; *n* = 3; *P < 0.05. *P*-values were calculated using an One-way ANOVA test. **P < 0.01; ***P < 0.001). The effect of the NLRP3-specific inhibitor shRNA on the virus copy numbers **(C)**, virus titers **(D)**, and levels of virus protein **(E)** in CSFV-infected cells. PBMCs were transfected with LtV-scramble or Ltv-NLRP3 for 24 h, followed by CSFV infection (MOI = 1). At 24 h pi and 48 h pi, the levels of CSFV mRNA, CSFV titers, and Npro protein levels were analyzed (mean ± SD; *n* = 3; *P < 0.05. *P*-values were calculated using an One-way ANOVA test. **P < 0.01; ***P < 0.001).

As a pattern recognition receptor, NLRP3 can identify a variety of viral and antiviral effects by activating the IL-1β pathway. This effect has been reported for influenza virus and HCV virus replication, however, a similar role for CSFV infection is unknown. In order to investigate the role of inflammation in CSFV replication, we interfered with NLRP3 in different ways that ultimately inhibited the expression of NLRP3 protein and promoted the replication of CSFV. Results from these studies demonstrated that NLRP3 elicited effects on CSFV (Figures 7A–E).

### Cell viability was not affected by glibenclamide or Ltv-NLRP3 shRNA

**Figure 8:**
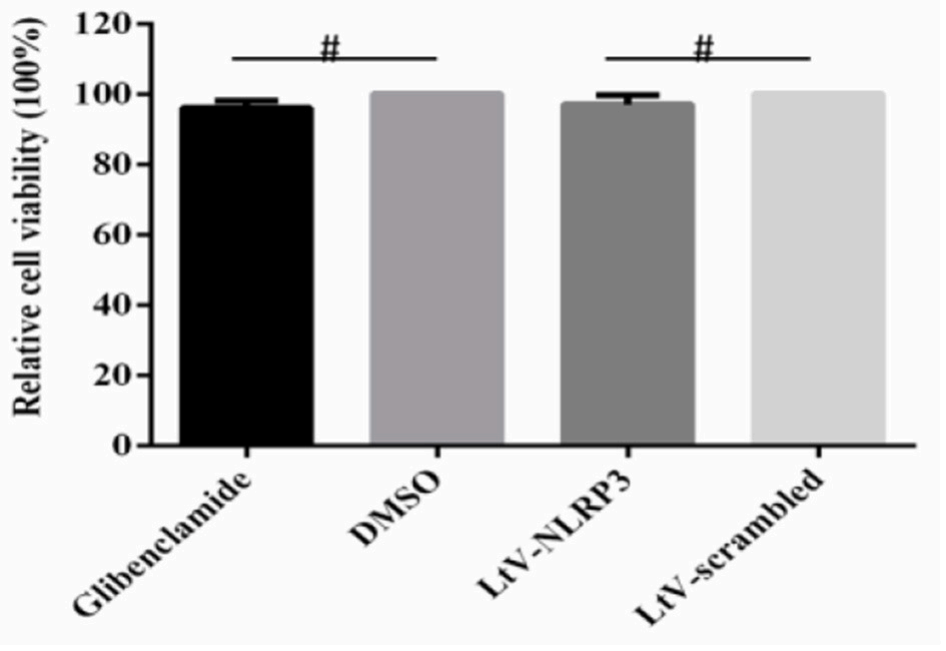
Effect of shRNA interference on cell viability. The viability of PBMCs treated with glibenclamide, DMSO, LtV-NLRP3, and LtV-scrambled shRNA was analyzed using the EthD-III/calcein AM staining assay (mean ± SD; *n* = 3; NSP > 0.05). ^#^*P* > 0.05. *P*-values were calculated using an One-way ANOVA test.

To verify that the inhibition of viral replication by glibenclamide and LtV-NLRP3 shRNAs was not caused by cell death, we used CCK-8 assays to determine the effect of glibenclamide and LtV-NLRP3 shRNA on PBMC activity. The results illustrated that glibenclamide and LtV-NLRP3 shRNA had no significant effect on the activity of PBMC cells (*P* > 0.05; Figure 8).

## Discussion

CSFV infection leads to host cell immune suppression but does not cause cytopathic effects or death in the cells (Bensaude et al., 2004; Johns et al., 2010). The mechanism by which CSFV persists in infected cells remains unclear (Knoetig et al., 1999; Blome et al., 2013). Previous studies have shown that CSFV can utilize autophagy or mitophagy to inhibit apoptosis and promote cell survival (Gou et al., 2017). Pyroptosis is another form of programmed cell necrosis and is also called cellular inflammatory necrosis (Shi et al., 2017). Pyroptosis is a programmed necrosis mediated by gasdermin and plays a critical part in the innate immune response and antiviral response to infection (Jorgensen and Miao, 2015; Shi et al., 2017). The activation of the inflammatory caspase 1 and caspase 11 regulates pyroptosis in order to avoid excessive damage to the host organism. The mechanism for how the cell death program is initiated by these caspases is not yet clear.

Pyroptosis has been reported to play an important role in the survival and replication of many viruses. In HIV infected patients, pyroptosis can induce the disappearance of CD4 T-cells. Approximately 5% of CD4 cell-death is induced by caspase 3-mediated apoptosis and the other 95% of CD4 T-cell death is induced by caspase 1-mediated pyroptosis.The activation of inflammasome promotes the occurrence of pyroptosis and the release of IL-1β, and the inflammatory signal that is released promotes additional CD4T cell death (Doitsh et al., 2014). HCV infection can also reduce the survival rates of cells by way of caspase 3-mediated apoptosis and inflammasome-activation of caspase 1, which mediates pyroptosis. Co-culture of HCV-infected Hch-75 cells and uninfected HCV cell lines leads to the death of the uninfected cells (Kofahi et al., 2016). In addition to the important role of the apoptotic assembly in viral infection, the role of apoptosis in other disease models is also significant. In an allergic mouse model, the production of IL-1β and the activity of caspase 1 are significantly increased compared with the control group, indicating that the activity of caspase 1 and the expression of IL-1β are closely related to the IPAF inflammasome (Mascarenhas et al., 2017). In BNP (B-type-natriuretic peptide), myocardial cell death is regulated by the up-regulation of LncRNA LSINCT5 and activation of the caspase 1/IL-1β signaling pathway (Zhang et al., 2015). However, the role of pyroptosis in CSFV infection has not been reported to date.

We observed that CSFV could promote the cleavage and enzyme activity of caspase 1 in PBMCs and induce pyroptosis. This result indicated that pyroptosis and apoptosis both have a vital function in CSFV replication and participate in the persistent infection of cells with CSFV. After the virus invades the body, the initial interaction between the host and the virus is the identification of the virus by the host cell, which underlies the basis of antiviral immunity (Di Paolo, 2014). The inflammasome pathway is a critical early-response mechanism by the host that enables the detection of pathogens and initiates the production of inflammatory cytokines, which induces the recruitment of effector cells to the site of infection (Martinon et al., 2002; Prochnicki et al., 2016). The inflammasome complex consists of intracellular multiprotein oligomers, which include a sensor protein such as the nucleotide binding oligomerization domain (NOD)-like receptor proteins (NLRP), and an adapter protein, ASC, which critically activates pro-caspase1 (Ranson et al., 2017). The mature caspase-1 then proteolytically cleaves cytosolic pro-IL-1β and pro-IL-18, which are subsequently secreted as inflammatory cytokines that activate the inflammatory arm of the immune response to infection (Edye et al., 2015). Activated caspase 1 also results in pyroptosis, which is a form of cell death that is triggered by inflammation (Malik and Kanneganti, 2017).

The induction and activation of IL-1β and IL-18 are considered to be critical signatures of inflammasome activation. NLRP3 is one of the key inflammasome molecules implicated in the identification of ssRNA and double-stranded DNA viruses. It has been demonstrated that infection with viral RNA and subsequent activation of the NLRP3 inflammasome is an important part of the host defense against IAV infection. The activation of the NLRP3 inflammasome during sub-lethal influenza infection plays a critical role in limiting lung damage resulting from infection (Allen et al., 2009). Therefore, the activation of the NLRP3 inflammasome represents a critical balance for the controlled production of the inflammatory cytokines IL-1β and IL-18, as excessive or prolonged stimulation adds to the inflammatory disease burden, whereas the appropriate activation enables efficient responses to the infection and clearance of viruses.

In a previous study, we have found that by binding to MDA5 and RIG-I, CSFV activates the RIG-I signaling pathway to induce the activation of NF-κB and IRF-3 nuclear translocation, thus triggering the production of antiviral and inflammatory cytokines (Dong et al., 2013). Apoptosis is the main defense mechanism when a virus infects the host (Aubert and Jerome, 2003; Koyama et al., 2003). Previous studies have reported that *in vitro* CSFV infection prevents cell death by interfering with IFN production. IFN production is related to the inhibition of IRF3, a key inducer of type I IFN expression by the viral Npro protein (Bauhofer et al., 2007). However, the effects of CSFV on NOD pattern recognition receptors and pyroptosis are still poorly understood.

**Figure 9:**
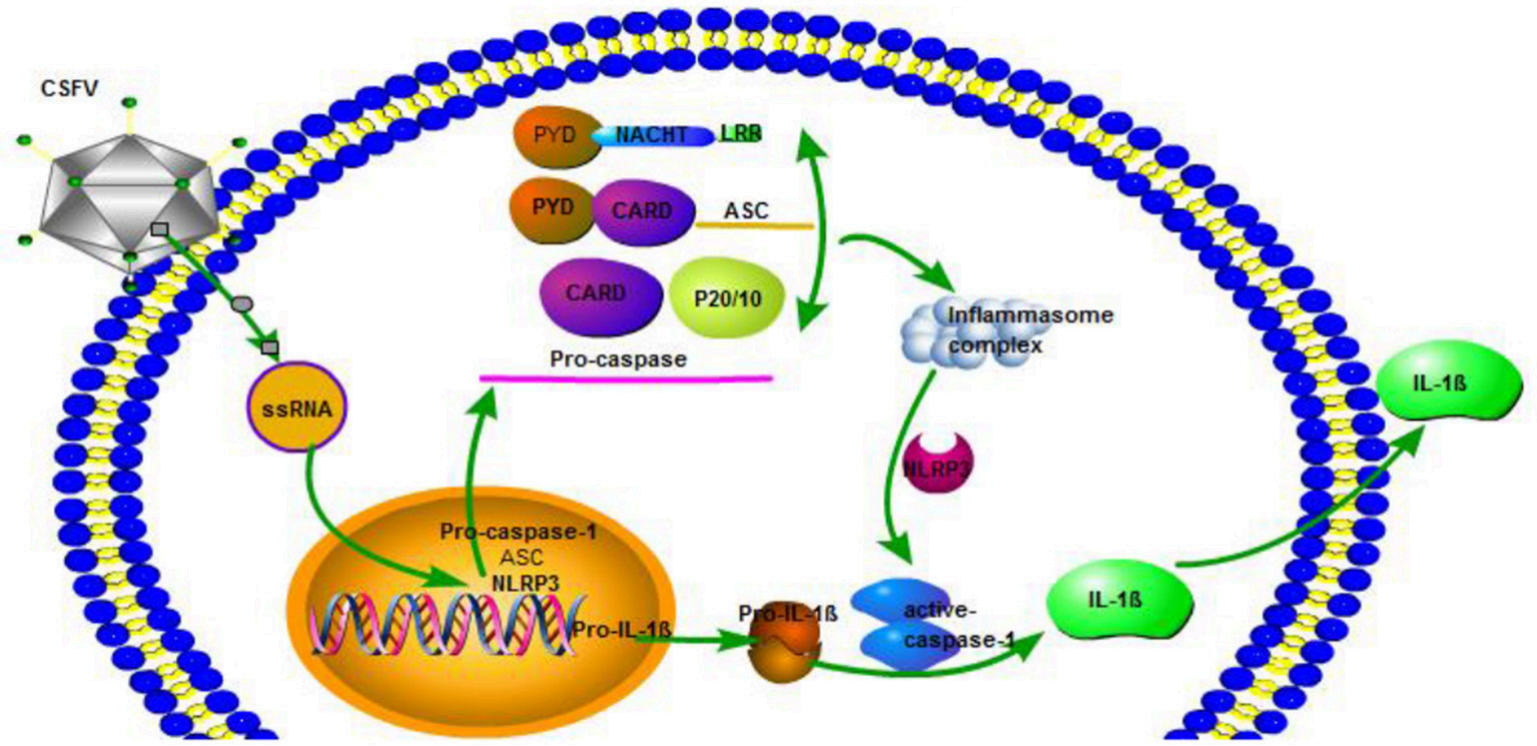
CSFV activation of interleukin-1β release is dependent on NLRP3. CSFV infection activates caspase-1, induces the formation of inflammatory complexes ASC, and activates caspase-1 through pro-IL-1β and NLRP3 to promote the release of IL-1β.

In the current study, we observed that the infection of CSFV could promote the release of IL-1β, and this effect reached the highest value after 24 and 36 h. At the same time, CSFV infection could promote the expression of pro-1b and IL-1β subunit p17 protein. Because the classical pathway of IL-1β release requires caspase1 activation, we explored whether CSFV promoted the release of IL-1β through caspase1. We observed that CSFV infection promoted the expression of caspase 1 and pro-caspase 1, as well as caspase 1 activity. In addition, treatment with the caspase 1 specific inhibitor Ac-yVAD-CHO, the activation of IL-1β induced by CSFV was inhibited. These results confirmed that CSFV infection promoted the release of IL-1β through caspase 1. We also confirmed that swine fever virus infection can promote PBMC pyroptosis. The inflammasome complex consists of intracellular multiprotein oligomers, which include a sensor protein, such as the nucleotide binding oligomerization domain (NOD)-like receptor protein (NLRP), the adapter protein ASC, and NLRP3. Inflammasome formation induced by ASC critically activates pro-caspase 1. In CSFV infection, this protein has important effects on the activation of caspase 1, K+ channel formation and ATP levels in monocytes. We found that the release of IL-1β induced by CSFV infection depended on the function of the NLRP3 inflammatory corpuscle and the RIG-I gene, and that CSFV infection inhibited the expression of NLRP3 and ASC in PBMCs. The use of glibenclamide and interference plasmids to inhibit NLRP3 inflammasome activation revealed that the number of CSFV virus gene copies and the virus titer after 24 and 48 h increased following CSFV infection in PBMCs, indicating that NLRP3 had a negative effect on CSFV replication. NLRP3 inflammatory bodies can activate caspase 1 in response to conserved structures and endogenous threatening signals of various pathogens and mediate the proinflammatory cytokine precursor pro-IL-1β. In addition, the maturation and secretion of pro-IL-18 activate immune defenses and play an important role in anti-microbial infection. After activation of NLRP3, self-oligomerization occurs and caspase 1 precursors are recruited via the linker protein ASC to assemble into NLRP3 inflammasomes, mediate activation of caspase 1, and promote the maturation of proinflammatory cytokines IL-1β and IL-18. This anti-infectious immunity of the NLRP3 inflammasome may be responsible for the restrict of CSFV (Figure 9). In an infection model for influenza virus, type I IFN initiates an anti-influenza virus effect through an RIG-I/TLR3/NLRP3 dependent pathway. NLRP3 recognizes the influenza virus through caspase 1 and plays a role in adaptive immunity.

In conclusion, our results provided evidence for the first time that CSFV utilizes caspase 1 and NLRP3 to enhance IL-1β production and activation. The induction of pyroptosis that may be mediated by CSFV-induced inflammasome in host cells, especially in PBMCs, may help explain why CSFV establishes a persistent infection in leukocytes. Monocytic cells play key roles in immune pathology; thus, viral particles parasitizing immune cells and interfering with their function may be the cause of immunosuppression due to CSFV. Overall, the aberrant IL-1β-release and pyroptosis observed in this study offer a distinct perspective on the pathogenesis of CSFV infection and suggest a novel approach in the development of antiviral strategies.

## Ethics statement

The authors declare that the animal breeding, care and all experiments were performed in adherence to the guidelines of the Laboratory Animal Center of South China Agricultural University and approved by the Animal Ethics Committee.

## Author contributions

SF, JC, and MiZ designed experiments; SF, JYu, SD, and YC carried out experiments; SF, BX, MeZ, and HX analyzed experimental results and data. KW, YH, YZ, and JYa assisted with animal experiment. SF and JYu wrote the manuscript.

### Conflict of interest statement

The authors declare that the research was conducted in the absence of any commercial or financial relationships that could be construed as a potential conflict of interest.
